# Comparative metagenomic and metatranscriptomic analyses reveal the breed effect on the rumen microbiome and its associations with feed efficiency in beef cattle

**DOI:** 10.1186/s40168-019-0618-5

**Published:** 2019-01-14

**Authors:** Fuyong Li, Thomas C. A. Hitch, Yanhong Chen, Christopher J. Creevey, Le Luo Guan

**Affiliations:** 1 Department of Agricultural, Food and Nutritional Science, University of Alberta, Edmonton, Alberta T6G 2P5 Canada; 2 The Centre of Excellence for Gastrointestinal Inflammation and Immunity Research (CEGIIR), University of Alberta, Edmonton, Alberta T6G 2E1 Canada; 3 Institute of Biological, Environmental and Rural Sciences (IBERS), Aberystwyth University, Aberystwyth, Wales SY23 3FG UK

**Keywords:** Rumen, Microbiome, Metagenomics, Metatranscriptomics, Feed efficiency, Beef breed

## Abstract

**Background:**

Microorganisms are responsible for fermentation within the rumen and have been reported to contribute to the variation in feed efficiency of cattle. However, to what extent the breed affects the rumen microbiome and its association with host feed efficiency is unknown. Here, rumen microbiomes of beef cattle (*n* = 48) from three breeds (Angus, Charolais, Kinsella composite hybrid) with high and low feed efficiency were explored using metagenomics and metatranscriptomics, aiming to identify differences between functional potentials and activities of same rumen microbiomes and to evaluate the effects of host breed and feed efficiency on the rumen microbiome.

**Results:**

Rumen metagenomes were more closely clustered together and thus more conserved among individuals than metatranscriptomes, suggesting that inter-individual functional variations at the RNA level were higher than those at the DNA level. However, while mRNA enrichment significantly increased the sequencing depth of mRNA and generated similar functional profiles to total RNA-based metatranscriptomics, it led to biased abundance estimation of several transcripts. We observed divergent rumen microbial composition (metatranscriptomic level) and functional potentials (metagenomic level) among three breeds, but differences in functional activity (metatranscriptomic level) were less apparent. Differential rumen microbial features (e.g., taxa, diversity indices, functional categories, and genes) were detected between cattle with high and low feed efficiency, and most of them were breed-specific.

**Conclusions:**

Metatranscriptomes represent real-time functional activities of microbiomes and have the potential to better associate rumen microorganisms with host performances compared to metagenomics. As total RNA-based metatranscriptomics seem to avoid potential biases caused by mRNA enrichment and allow simultaneous use of rRNA for generation of compositional profiles, we suggest their use for linking the rumen microbiome with host phenotypes in future studies. However, if exploration of specific lowly expressed genes is desired, mRNA enrichment is recommended as it will enhance the resolution of mRNA. Finally, the differential microbial features observed between efficient and inefficient steers tended to be specific to breeds, suggesting that interactions between host breed genotype and the rumen microbiome contribute to the variations in feed efficiency observed. These breed-associated differences represent an opportunity to engineer specific rumen microbiomes through selective breeding of the hosts.

**Electronic supplementary material:**

The online version of this article (10.1186/s40168-019-0618-5) contains supplementary material, which is available to authorized users.

## Background

Beef cattle are both an important source of high quality protein (meat) and economic stability for humans. With the increase in global human population, there is increased competition for resource (e.g., land, water, and cereal grains) between human and livestock, especially for beef cattle operations [1, 2]. Improving cattle feed efficiency would enhance the feed utilization ratio, thus reducing the amount of feed consumed (especially human edible cereal grains) while maintaining higher or equal production performance. Additionally, cattle with high feed efficiency not only emit less CH_4_ (~ 25%), but also excrete less feces than cattle with low feed efficiency [3, 4]. Therefore, improving feed efficiency can also decrease the negative environmental effects caused by beef cattle operations.

The rumen microbiota consists of bacterial, archaea, fungi, ciliated protozoa, and phages [5], which are responsible for the rumen fermentation. Several studies have revealed their associations with feed efficiency in beef and dairy cattle [6–11], reporting the differences in relative abundance of several rumen microbial phylotypes between efficient and inefficient individuals [6–10]. In addition, alpha-diversity indices of rumen bacterial and archaeal communities have also been reported to contribute to the variation in feed efficiency of cattle, where inefficient individuals possessed more complex and diverse microbial communities [10, 11]. However, most of these studies mentioned above only focused on the taxonomic profiles, and the linkages between rumen microbial metabolic functions and feed efficiency have not yet been well defined.

Metagenomics and metatranscriptomics have become powerful tools to estimate the functional potentials (DNA-based) and functional activities (RNA-based) of the rumen microbiome, which were comprehensively reviewed recently [12]. Because of the low proportion of mRNA in total rumen microbial RNA (usually < 10%) [13, 14], an mRNA enrichment step is normally conducted prior to library construction [15–17] to increase the sequencing depth of mRNA and capture more transcripts. Among the different strategies for prokaryotic mRNA enrichment, rRNA depletion based on subtractive hybridization using commercial kits [18] is one of the most widely applied approaches, not only for rumen samples [16, 17] but also for other types of environmental samples [19, 20]. Early studies stated this mRNA enrichment strategy did not significantly affect the estimation of metatranscriptomic profiles in synthetic bacteria mixtures [21, 22] or human fecal samples [23]; however, it is not currently known whether this holds for other microbial sample types such as from the rumen. An alternative approach which involves the sequencing of the total RNA without mRNA enrichment has been shown to successfully generate functional profiles for the rumen microbiome [14, 24] and provides an opportunity to test if mRNA enrichment causes any biases in this environment.

To date, there have been few studies applying a combined meta-omics approach to dissect the functional potentials and activities of the rumen microbiome and its role in host cattle feed efficiency. Two recent studies which linked rumen microbial functional profiles to feed efficiency in cattle used either metagenomics [11] or metatranscriptomics [24] in isolation. These studies suggested that rumen microbiomes of inefficient cattle may have more diverse functional potentials in dairy [11] and higher activities in beef [24] cattle than those in efficient cattle, leading to a wider range of fermentation products. Ideally, we would like to know if these products are efficiently utilized and/or even harmful to the host; however, none of these existing studies has considered the role of host genetic background.

Previous studies have shown that rumen microbial taxonomic profiles were distinguishable among hosts with different genetic backgrounds [25, 26]. This could partially explain why association patterns between the rumen microbiome and feed efficiency show low consistency across studies [6–10]. In addition, diet has been shown to be the major factor affecting the rumen microbial community [25], and rumen microbiota are distinct between forage-fed and concentrate-fed animals [27, 28]. Furthermore, repeated measurements of feed efficiency of the same animals under both forage- and concentrate-based diets has been shown to result in changes in efficiency ranking in over 50% of the cattle examined [29], suggesting that diet must be consistent across all studied animals if the breed effect on the rumen microbiome and linkages between the rumen microbiome and feed efficiency are to be precisely estimated. Therefore, in the present study, rumen microbiomes of beef cattle from three different breeds receiving the same diet but with variations in high and low feed efficiency were explored using metagenomics, total RNA-based metatranscriptomics, and mRNA-enriched metatranscriptomics, aiming to evaluate the breed effect on the rumen microbiome and to generate more conclusive understanding of the role of the rumen microbiome in beef cattle feed efficiency. In addition, the direct comparison between mRNA-enriched and total RNA-based metatranscriptomics for the same samples was conducted to provide useful information for future rumen metatranscriptomic study design.

## Methods

### Animal experiments and sample collection

Forty-eight steers were selected from a herd of 738 beef cattle that were born in 2014 and raised at the Roy Berg Kinsella Research Ranch, University of Alberta, according to their breeds and residual feed intake (RFI) ranking. These 48 steers belong to three breeds and two RFI groups (high RFI [H-RFI, inefficient] and low RFI [L-RFI, efficient]), including two purebreds (Angus [ANG]; H-RFI, *n* = 8; L-RFI, *n* = 8) and Charolais [CHAR]; H-RFI, *n* = 8; L-RFI, *n* = 8), and one crossbred (Kinsella composite hybrid [HYB]; H-RFI, *n* = 8; L-RFI, *n* = 8). The animal study was approved by the Animal Care and Use Committee of the University of Alberta (no. AUP00000882), following the guideline of the Canadian Council on Animal Care [30]. The HYB population was bred from multiple beef breeds including Angus, Charolais, Galloway, Hereford, Holstein, Brown Swiss, and Simmental as described previously [31]. These animals were all under the same feedlot condition and fed with the same high-energy finishing diet which consisted of 80% Barley grain, 15% Barley silage, and 5% Killam 30% Beef Supplement Pellets (Tag 849053; Hi-Pro Feeds, Westlock, AB, Canada). Dry matter intake (DMI) and eating frequency (times of an individual visiting the feed bunk per day) were individually recorded using the GrowSafe system (GrowSafe Systems Ltd., Airdrie, AB, Canada). RFI values were calculated based on DMI, average daily gain (ADG), metabolic weight (MWT), and back fat thickness as descried previously [32]. Steers were slaughtered before feeding at Lacombe Research Centre (Agriculture and Agri-Food Canada, Lacombe, AB, Canada). Rumen digesta samples were collected at slaughter, snap-frozen using liquid nitrogen, and stored under − 80 °C until further analysis. Rumen weight was obtained after completely emptying rumen digesta and fluid using a weight balance.

### DNA extraction and metagenome sequencing

Total genomic DNA was isolated from rumen digesta using the repeated bead beating plus column (RBB + C) method as described in [33]. The quality and quantity of DNA was measured using a NanoDrop Spectrophotometer ND-1000 (Thermo Fisher Scientific Inc., Wilmington, DE, USA). Metagenome library was constructed using the TruSeq DNA PCR-Free Library Preparation Kit (Illumina, San Diego, CA, USA), and the quantity of each library was evaluated using a Qubit 2.0 fluorimeter (Invitrogen, Carlsbad, CA, USA). Sequencing of metagenome libraries was conducted at the McGill University and Génome Québec Innovation Centre (Montréal, QC, Canada) using Illumina HiSeq 2000 (100 bp paired-end sequencing of ~ 350 bp inserts).

### RNA extraction and metatranscriptome sequencing

Total RNA was extracted from rumen disgesta following the procedure described in [13]. The RNA yield was measured using a Qubit 2.0 fluorimeter (Invitrogen), and the RNA quality was measure using an Agilent 2200 TapeStation (Agilent Technologies, Santa Clara, CA, USA). Only samples with RNA integrity number (RIN) ≥ 7.0 were used to generate metatranscriptome libraries. In the current study, two types of metatranscriptome libraries were constructed: total RNA-based metatranscriptome libraries (T-metatranscriptome) and mRNA-enriched metatranscriptome libraries (M-metatranscriptome). For the M-metatranscriptome library construction, rRNA in each sample was depleted using the Ribo-Zero Gold rRNA Removal Kit (Epidemiology) (Illumina) according to the manufacturer’s instruction. Total RNA and enriched mRNA were used for T- and M-metatranscriptome library construction, respectively, using the TruSeq RNA Library Prep Kit v2 (Illumina). Sequencing of T- and M-metatranscriptome libraries was conducted at the McGill University and Génome Québec Innovation Centre (Montréal, QC, Canada) using Illumina HiSeq 2000 (100 bp paired-end sequencing of ~ 140 bp inserts) and 2500 (125 bp paired-end sequencing of ~ 140 bp inserts), respectively.

### Analysis of metagenomes and metatranscriptomes

The quality control (QC) of each dataset was performed using Trimmomatic (version 0.35) [34] to trim artificial sequences (adapters), cut low quality bases (quality scores < 20), and remove short reads (< 50 bp). The program SortMeRNA (version 1.9) [35] was used to extract rDNA and rRNA reads from sequencing datasets. Non-rDNA/rRNA reads were then mapped to the bovine genome (UMD 3.1) using Tophat2 (version 2.0.9) [36] to remove potential host DNA and RNA contaminations. Taxonomic profiles of the active rumen microbiota were generated using 16S rRNA extracted from T-metatranscriptomes following the pipeline described in [13]. Briefly, post-QC bacterial and archaeal 16S rRNA reads were aligned to the V1-V3 region-enriched Greengenes database (version gg_13_8) [37] and the V6-V8 region-enriched RIM-DB database [38], respectively. After that, mapped reads were taxonomically classified using the naive Bayesian approach [39] in mothur [40].

To estimate rumen microbial functional profiles, non-rDNA sequences from all metagenomes (*n* = 48) were pooled, assembled, and annotated to create a functional reference database. Briefly, the pooled metagenomes were de novo assembled using Spherical program [41]. Within Spherical, Velvet [42] was set as the assembler with the kmer size of 31, Bowtie2 [43] was set as the aligner, and 25% of total pooled sequences were subsampled as the input for each iteration of assembly with eight iterations in total. After the de novo assembly of pooled metagenomes reads, a total of 57,696,422 contigs with an average length of 144 bp (max 135,846 bp) and a N50 length of 140 bp were generated. Assembled contigs were then annotated using the blastx module in DIAMOND [44] against the UniProt database [45], and only annotations with bitscore > 40 were kept for the downstream analysis. Overlapped annotations were filtered and converted to the GFF format using the MGKit package (https://bitbucket.org/setsuna80/mgkit). After discarding short contigs with length < 60 bp, 20,314,713 contigs (35.21%) were successfully annotated with an average length of 195 bp and a N50 length of 197 bp. To identify the functional categories of metagenomes, T-metatranscriptomes, and M-metatranscriptomes, non-rDNA/rRNA sequences were individually aligned to above annotated contigs using Bowtie2 and then were counted using HTSeq [46]. Only reads mapped to contigs with eggNOG annotation information [47] were further retrieved to calculate the abundances of genes and functional categories using MGKit.

### Statistical analysis

Values of RFI, DMI, eating frequency, and rumen weight were compared among three breeds using ANOVA, and the comparison between efficient (L-RFI) and inefficient (H-RFI) animals were conducted using *t* test within each breed separately. In the current study, only microbial taxa with a relative abundance higher than 0.01% in at least 50% of individuals within each breed were considered as being observed and used for the analysis. Bacterial compositional profiles were summarized at phylum and genus levels, and archaeal communities were summarized at the species level. Relative abundances of microbial taxa were arcsine square root transformed [19, 24], and then compared among breeds (using ANOVA) and between RFI groups within each breed (using *t* test). To make alpha-diversity indices (including Chao1, Shannon evenness, Simpson evenness, Shannon index, and inverse Simpson) comparable among samples, the number of sequences per sample was normalized to the lowest reads number for bacteria (*n* = 274,885) and archaea (*n* = 4263), respectively. These indices were compared between H- and L-RFI groups within each breed using Kruskal-Wallis rank-sum test. Principal coordinate analysis (PCoA) was used to visualize rumen microbial communities based on the Bray-Curtis dissimilarity matrices at the genus level for bacteria and at the species level for archaea.

Only functional categories and genes/transcripts with a minimum relative abundance of 0.01% in at least three samples within a dataset were considered as being detected as suggested in [19]. The abundance of each gene/transcript was then normalized into counts per million (cpm). To compare general microbial functional profiles among different datasets, breeds, and RFI groups, a principal component analysis (PCA) was conducted based on the auto-scaled cpm of functional categories and genes (or transcripts). Correlations between datasets were calculated using Spearman’s rank correlation. Differential abundances of functional categories and genes (or transcripts) were compared among sequencing datasets, breeds, and RFI groups using DESeq2 [48].

## Results and discussion

RFI values were not significantly different among the three beef cattle breeds (*P* = 0.73), but they were significant different between L- and H-RFI animals within each breed (*P* < 1.00e−5; Table 1). Following quality control, a total of 2622.07 M, 3087.41 M, and 2645.13 M sequences were generated from the metagenomes (54.63 ± 1.42 M; per sample mean ± SEM), T-metatranscriptomes (64.32 ± 0.74 M), and M-metatranscriptomes (55.11 ± 1.90 M), respectively. From metagenomes/T-/M-metatranscriptomes, 99.37 ± 0.03%/6.29 ± 0.16%/53.34 ± 2.14% (mean ± SEM) sequences were classified as non-rDNA/rRNA, and sequences aligned to the bovine genome were lower than 0.20% in all three datasets (Table 2).

**Table 1:** Phenotypes of three beef breeds used in the present study

	Angus (*n* = 16)	Charolais (*n* = 16) (mean ± SEM)	Kinsella composite hybrid (*n* = 16)	*P* value^1^
Residual feed intake (RFI; kg/day)				
Overall	0.15 ± 0.21	0.06 ± 0.24	− 0.10 ± 0.24	0.73
L-RFI (*n* = 8)	− 0.58 ± 0.10	− 0.81 ± 0.10	− 0.96 ± 0.10	
H-RFI (*n* = 8)	0.88 ± 0.15	0.92 ± 0.12	0.76 ± 0.13	
*P* value^2^	1.46e−06	1.87e−08	6.43e−08	
Dry matter intake (DMI; kg/day)				
Overall	10.73 ± 0.27^a^	10.33 ± 0.28^a^	9.27 ± 0.28^b^	3.23e−03
L-RFI (*n* = 8)	10.19 ± 0.34	9.49 ± 0.24	8.98 ± 0.34	
H-RFI (*n* = 8)	11.35 ± 0.31	11.17 ± 0.29	9.67 ± 0.43	
*P* value^2^	0.03	5.80e−04	0.23	
Eating frequency (*n*/day)				
Overall	37.63 ± 1.61^a^	36.59 ± 1.56^a^	29.73 ± 1.99^b^	6.30e−03
L-RFI (*n* = 8)	41.25 ± 1.53	35.56 ± 2.65	29.94 ± 2.02	
H-RFI (*n* = 8)	33.49 ± 2.13	37.63 ± 1.77	29.44 ± 4.18	
*P* value^2^	9.90e−03	0.53	0.91	
Empty rumen weight (kg)				
Overall	13.02 ± 0.59^a^	11.29 ± 0.29^b^	NA	1.36e-02
L-RFI (*n* = 8)	12.42 ± 0.80	11.15 ± 0.38	NA	
H-RFI (*n* = 8)	13.62 ± 0.87	11.43 ± 0.47	NA	
*P* value^2^	0.33	0.65	NA	

*NA* not available

^1^*P* values among three breeds were calculated using ANOVA, and values with different superscripts were significantly different (*P* < 0.05)

^2^*P* values between H- and L-RFI groups were obtained using *t* test within each breed

**Table 2:** Summary of metagenome and metatranscriptome datasets

	Metagenome (*n* = 48)	T-metatranscriptome (*n* = 48) (mean ± SEM)	M-metatranscriptome (*n* = 48)
After QC	54.63 ± 1.42 M	64.32 ± 0.74 M	55.11 ± 1.90 M
non-rDNAs/rRNAs	99.37 ± 0.03%	6.29 ± 0.16%	53.34 ± 2.14%
rDNAs/rRNAs	0.63 ± 0.03%	93.71 ± 0.16%	46.66 ± 2.14%
Host DNAs/RNAs	0.13 ± 0.06%	0.05 ± 0.01%	0.14 ± 0.05%
Reads mapped back contigs	78.47 ± 0.26%	66.85 ± 0.65%	54.43 ± 1.16%
Reads mapped back annotated contigs	62.02 ± 0.56%	33.04 ± 0.54%	32.19 ± 1.50%
eggNOG annotated reads	1,010,497 ± 32,603	23,590 ± 1494	412,875 ± 30,166

*QC* quality control

The sequencing depth of our metagenomes is comparable with the rumen metagenomes published recently, which obtained assembly of 913 near-complete and draft bacterial and archaeal genomes [49]. Furthermore, similar sequencing depth has also been used in two pioneering studies to link the rumen metagenome with the phenotype of cattle [11, 50]. To further check whether our metagenomes have sufficient coverage, the metagenomic sequencing data of three samples (IDs: 101, 103, and 104) were selected and twice randomly subsampled at 50% to generate two subsamples for each sample. Each subsample was aligned to the assembled and annotated contigs, and more than 97.5% of observed genes within each sample could be detected by both subsamples, suggesting that even half size of our metagenomes could cover most of rumen microbial genes. Therefore, we believe that our metagenomes have sufficient coverage to represent the majority of microbial genomes in the bovine rumen.

### General functional profiles of the rumen microbiome at DNA and RNA levels

After filtering overlapped annotations, 20,314,713 contigs (35.21%) from pooled metagenomes were successfully annotated based on the UniProt database. An average of 62.02 ± 0.56%, 33.04 ± 0.54%, and 32.19 ± 1.50% sequences from metagenomes, T-metatranscriptomes, and M-metatranscriptomes could be mapped back to these annotated contigs, respectively. The ratio of mapped metagenome reads to annotated genes (62.02%) is comparable with a recent rumen metagenomic study on dairy cattle (52.40%) [11], indicating that around 40–50% rumen microbial genes have not been captured in current public databases. To determine the necessity of using assembled metagenome contigs as the reference dataset for the downstream analysis, three samples (IDs: 101, 103, and 104) were selected to compare outcomes between this assembly-based approach and the assembly-free approach (mapping reads to the UniProt database directly using DIAMOND). Through the assembly-free approach, 22.53 ± 0.52% of metagenome reads and 11.78 ± 2.33% of T-metatranscriptome reads could be mapped back to the UniProt database with an *E* value of 1e−5 as the cutoff. These ratios are much lower than those based on the assembly-based approach (57.56 ± 1.20% and 28.99 ± 0.27% for metagenome and T-metatranscriptome reads, respectively; *P* < 0.05, paired sample *t* test). For M-metatranscriptomes, 33.18 ± 2.49% reads could be mapped using the assembly-free approach, which is similar with the assembly-based method (33.25 ± 2.57%; *P* = 0.94), indicating that assembly-free approaches may be sufficient for mRNA enriched metatranscriptomic data. These results suggest that using the assembled metagenome contigs as the reference in our study was likely to be the best approach to maximize the capture of the functional capacity of the metagenome and T-metatranscriptome, while not impairing the results from the M-metatranscriptome.

Of the metagenome contigs, 3,589,489 were annotated with eggNOG information, and only reads mapped to these contigs were used to estimate functional profiles. Detailed information of sequencing datasets is listed in Table 2. In addition to eggNOG, several other databases, such as KEGG [51] and Gene Ontology (GO) [52], have been previously used for functional classification of rumen metagenomes and/or metatranscriptomes [15, 50, 53]. It has been suggested through the use of the MG-RAST server [54] on a rumen metagenomic dataset (ID: mgm4547164.3) that the use of different databases for functional annotation can lead to inconsistent numbers of annotated contigs and distinct types of annotation profiles [11]. For instance, a higher number of contigs were annotated based on KEGG than eggNOG or GO databases [11]. Similar results were also obtained for rumen metatranscriptome contigs from our previous study [24] through MG-RAST (ID: mgm4723666.3), suggesting the importance of database selection on rumen metagenomics and metatranscriptomics. In the absence of a gold standard dataset to compare, it is not possible to assess the false positive or negative rates of each functional annotation approach, and so it is more important that within any single study the annotation approaches are consistent to allow comparisons. Further comparison studies are required to assess the impact of database selection on rumen metagenomic and metatranscriptomic annotation.

**Fig. 1:**
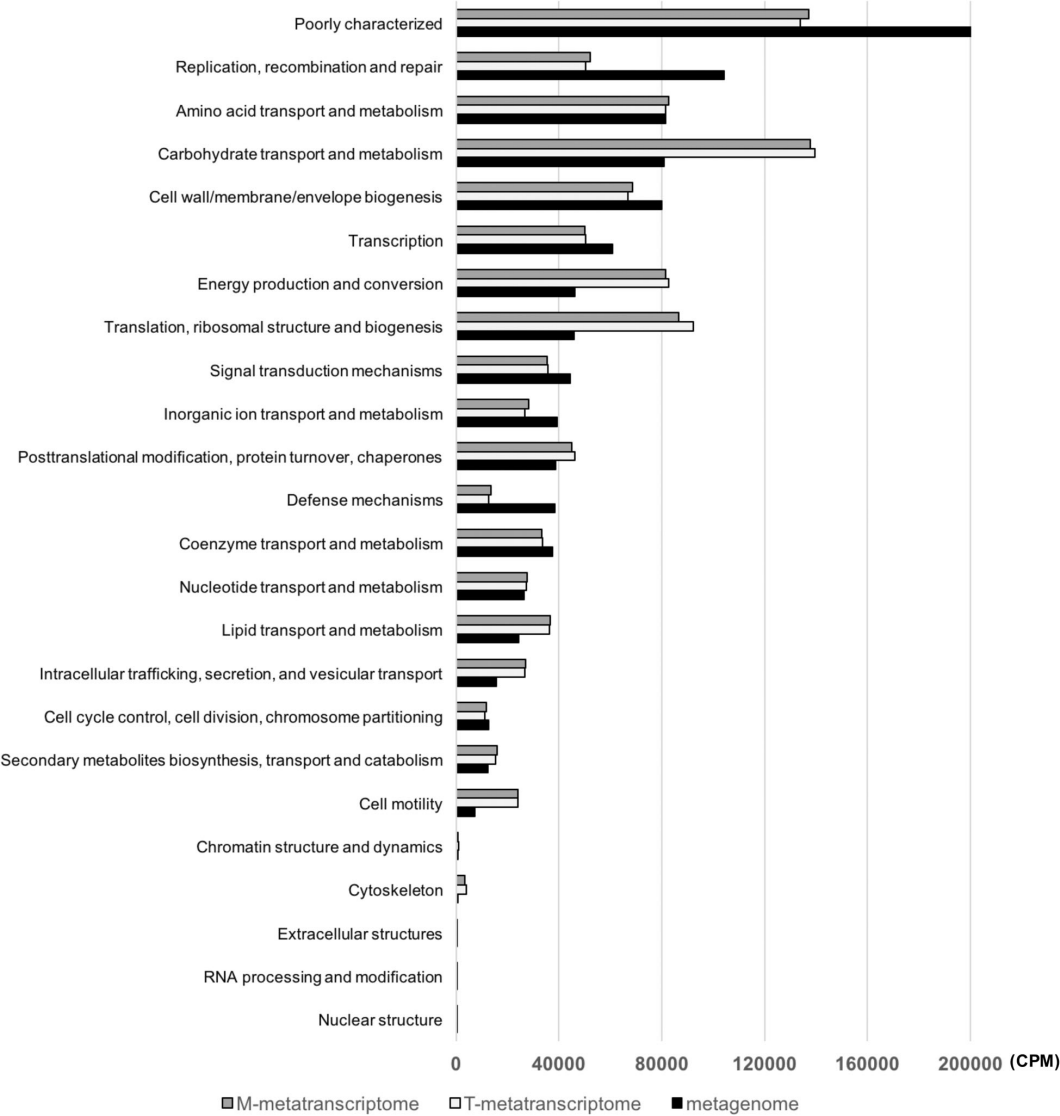
Abundances of observed eggNOG functional categories among metagenome, T-metatranscriptome, and M-metatranscriptome datasets. T- and M-metatranscriptome represents total RNA-based and mRNA-enriched metatranscriptome, respectively

In total, 23 eggNOG functional categories were observed through the functional analysis at both DNA and RNA levels. For the metagenomic data, 10.43%, 8.15%, and 8.10% were involved in “Replication, recombination and repair,” “Amino acid transport and metabolism,” and “Carbohydrate transport and metabolism,” respectively, and 20.07% were poorly characterized. For both T- and M-metatranscriptomes, “Carbohydrate transport and metabolism” was the most active functional category (13.96% and 13.78% in T- and M-metatranscriptomes, respectively), followed by the functional category of “Translation, ribosomal structure and biogenesis” (9.22% and 8.66% in T- and M-metatranscriptomes, respectively) (Fig. 1). This suggests that the majority of the functional activities in the rumen microbiome at the point when the digesta samples were collected were involved in replication, growth, and fermentation.

### Comparison between metagenomes and metatranscriptomes

**Fig. 2:**
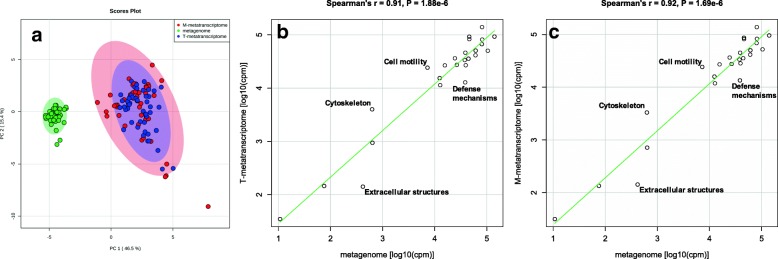
Distinguishable microbial functional profiles between rumen metagenome and metatranscriptome datasets. **a** PCA for eggNOG functional categories, which was calculated based on auto-scaled abundances (cpm) of functional categories. **b** Correlation between metagenome and T-metatranscriptome. **c** Correlation between metagenome and M-metatranscriptome. Each scatterplot in **b** and **c** illustrates log_10_-transformed mean abundances (cpm) of each functional category at DNA and RNA levels

The principle component analysis (PCA) based on eggNOG functional categories showed clear separation between metagenome and metatranscriptome functional profiles (Fig. 2a). Compared with T- and M-metatranscriptomes, metagenomes from rumen digesta samples were more closely clustered together and conserved among individuals (Fig. 2a), suggesting that inter-individual functional variations at the RNA level were higher than those at the DNA level. Therefore, rumen microbiomes from different individuals may have similar functional genetic potentials (at the DNA level), while their actual functional activities (at the RNA level) are noticeably more variable, similar to findings from the human gut microbiome [19].

Several functional categories were abundant at the DNA level but were more lowly expressed at the RNA level, such as “Replication, recombination and repair” (~ 2-fold, *P* < 0.05), “Extracellular structures” (~ 3-fold, *P* < 0.05), and “Defense mechanisms” (~ 3-fold, *P* < 0.05) (Fig. 1 and Fig. 2b, c). These functional categories may represent large functional potentials under environmental challenges. For instance, “Defense mechanisms” were downregulated in both T- and M-metatranscriptomes; however, their high abundances at the DNA level suggest that they could be activated in response to adverse conditions in the rumen, such as dietary change or an abrupt change in pH. Meanwhile, some functional categories including “Carbohydrate transport and metabolism,” “Translation, ribosomal structure and biogenesis,” “Cell motility,” and “Cytoskeleton” were highly expressed at the RNA level compared to their DNA abundances (2–6-fold, *P* < 0.05). The most active category was “Carbohydrate transport and metabolism” which is consistent with the rumen metatranscriptome analyses reported previously [24], indicating most of active microorganisms were fermenting carbohydrates (e.g., cellulosic plant materials and starch) when the digesta samples were collected.

Although general functional profiles were different between DNA and RNA levels, strong correlations were detected between metagenomes and metatranscriptomes (Spearman’s *r* = 0.91, *P* = 1.88e−6 between metagenomes and T-metatranscriptomes; *r* = 0.92, *P* = 1.69e−6 between metagenomes and M-metatranscriptomes; Fig. 2b, c), which is in line with the correlation patterns observed between human gut metagenomes and metatranscriptomes [19]. Through the linear regression estimation, metagenomes could explain 57.57% (*P* = 2.61e−06) and 60.81% (*P* = 6.67e−06) of variations in T- and M-metatranscriptomes, respectively. These strong correlations suggest that, as may be expected, gene expression profiles in the rumen microbiome are highly dependent on their gene abundances, even though other factors (such as environmental factors and post transcriptional regulation) also contribute to microbial gene expression variations in the rumen. To date, most existing associations reported between the rumen microbiome and host phenotypes (e.g., feed efficiency and methane emissions) are based on DNA data [7, 11, 50, 55]. It has been reported that differences in rumen microbial gene expression profiles, rather than genomic profiles, are associated with the variation in CH_4_ emissions of sheep [16]. Collectively, host phenotypic performances may be more associated with rumen microbial activities (at RNA level) than functional genetic potentials (at DNA level), and thus analysis at the RNA level may represent a more appropriate approach to link the rumen microbiome to host performances.

### Comparison between M- and T-metatranscriptomes

The mRNA enrichment step significantly removed rRNA from total RNA. There was 93.71 ± 0.16% rRNA in T-metatranscriptomes but only 46.66 ± 2.14% rRNA in M-metatranscriptomes (*P* = 7.36e−26; paired sample *t* test; Table 2), indicating a successful rRNA removal using the Ribo-Zero Gold rRNA Removal Kit. It is worth mentioning that the majority of the remaining rRNA in M-metatranscriptomes was classified as eukaryotic 28S rRNA (34.08 ± 2.29%), likely because the rRNA removal kit used is designed to hybridize and remove prokaryotic rRNA rather than eukaryotic rRNA. Therefore, the efficiency of mRNA enrichment using the Ribo-Zero kit is likely strongly affected by the microbial composition. A higher proportion of T-metatranscriptome reads could be mapped back to assembled metagenome contigs than M-metatranscriptome reads (66.85 ± 0.65% versus 54.43 ± 1.16%, *P* = 6.42e−18; paired sample *t* test). This suggests that T-metatranscriptomes are more similar to metagenomes, while M-metatranscriptomes may capture a greater number of more lowly expressed genes. Supporting this, we observed more eggNOG annotated mRNA reads in M- than in T-metatranscriptomes (412,875 ± 30,166 vs 23,590 ± 1494, *P* = 1.16e−17; paired sample *t* test).

**Fig. 3:**
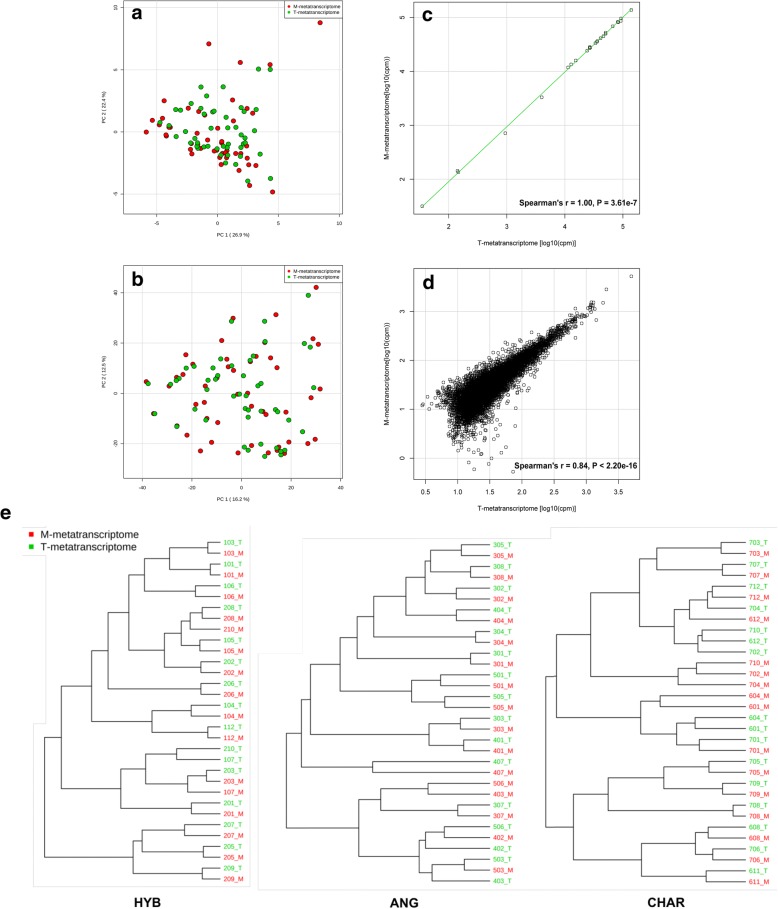
Microbial functional profiles of T- and M-metatranscriptomes. PCA for eggNOG functional categories (**a**) and expressed genes (**b**), which were performed based on auto-scaled abundances (cpm) of functional features. Correlations between rumen T- and M-metatranscriptomes were calculated using Spearman’s rank correlation based on functional categories (**c**) and expressed genes (**d**). Each scatterplot in **c** and **d** illustrates log_10_-transformed mean abundances (cpm) of each functional category and each expressed gene. **e** Cluster analysis showing that between-method variations were lower than between-subject variations, which was conducted based on auto-scaled abundances (cpm) of functional categories using Euclidean as distance measure and Ward as clustering method

According to the PCA, overall functional profiles did not show clear difference between T- and M-metatranscriptomes (Fig. 3a, b). At the same time, strong correlations were detected between T- and M-metatranscriptomes based on both functional categories (Spearman’s *r* = 1.00, *P* = 3.61e−7) and expressed genes (Spearman’s *r* = 0.84, *P* < 2.20e−16) (Fig. 3c, d). The linear regression analysis based on functional categories and expressed genes gave *R*^*2*^ value of 1.00 (*P* < 2.2e−16) and 0.94 (*P* < 2.2e−16), respectively, when compared T- with M-metatranscriptomes, confirming that T- and M-metatranscriptomes were highly similar to each other. When the cluster analysis was performed within each breed, T- and M-metatranscriptomes from the same sample were similar (between-method variations < between-subject variations), except for a few samples (Fig. 3e). These results are consistent with previous studies that compared metatranscriptomes between mRNA-enriched (based on the rRNA depletion) and total RNA-based libraries for synthetic bacteria mixture [21, 22] and human stool [23]. This suggests that the rRNA depletion based on subtractive hybridization using commercial kit (e.g. Ribo-Zero) did not significantly skew the general expression profile of rumen microbiomes, and thus individual variations among subjects could be estimated using both total RNA and enriched mRNA.

However, when abundance of each functional category was compared between T- and M-metatranscriptomes using the DESeq2 analysis, ten differential abundant functional categories (*P* < 0.05) were identified, even though their fold changes were low (from − 1.32 to 1.06; Fig. 3c). At the same time, the DESeq2 analysis revealed that 2050 genes had different abundances between T- and M-metatranscriptomes (FDR < 0.05), and most of them were underestimated in M-metatranscriptomes (Fig. 3d). In line with our results, a previous study using the same rRNA depletion method also detected the decreased abundances of several transcripts, which were considered as residual rRNA and/or transcripts from genes overlapped with rRNA genes [23]. However, in the current study, because putative rRNA transcripts have been removed through the program SortMeRNA (see the “Methods” section), it is reasonable to speculate that the underestimation of many expressed genes in M-metatranscriptomes may be caused by the mRNA degradation during the extended sample processing time.

According to our results, although applying mRNA enrichment could increase the sequencing depth of mRNA and enhance the resolution of metatranscriptomics on the functional analysis, it may bring about biases for the estimation of gene expression levels. In contrast, total RNA-based metatranscriptomics not only generates similar functional profiles as mRNA-enriched metatranscriptomics, but also can be used for the taxonomic identification [13]. Considering the rapid reduction of NGS costs, plus our current findings described above, total RNA sequencing rather than enriched-mRNA sequencing is to be recommended for global screening of the compositional and functional characteristics of the rumen microbiome and for linking with host phenotypes. However, because of the low proportion of putative mRNA in T-metatranscriptomes (6.29 ± 0.16%), a minimum sequencing depth should be determined to allow for sufficient coverage of expressed genes in the rumen microbiome. In the present study, six T-metatranscriptome libraries were mixed and sequenced in one lane of Illumina HiSeq 2000. Due to the higher sequencing depth for mRNA in M-metatranscriptome datasets (~ 17.5-fold higher than T-metatranscriptome datasets; Table 2), capture of more lowly expressed rumen microbial genes is possible. Therefore, if specific genes and/or metabolic pathways with low expression levels are required, mRNA enrichment is recommended for the enhanced resolution of mRNA.

### Compositional profiles of the active rumen microbiota

From T-metatranscriptomes, a total of 38,610,728 sequences were identified as representing the bacterial V1–V3 region of the 16S rRNA (804,390 ± 63,802; mean ± SEM) and 745,816 sequences from the archaeal V6–V8 region of the 16S rRNA (15,538 ± 1388). Each of these was used to generate taxonomic profiles of active rumen bacterial and archaeal communities. It is notable that there were only 42.15% and 64.39% bacterial and archaeal sequences falling within named genera and named species, respectively (Additional file 1: Table S1). The high proportion of unclassified taxa at the deep taxonomic level emphasizes that more effort is necessary to comprehensively characterize rumen microorganisms, especially to expand the coverage of rumen microbial genomes in current databases. In the present study, to better represent rumen microbial communities and detect potential associations between microbial taxa and feed efficiency, unnamed and/or unclassified taxa were included in the analysis.

**Fig. 4:**
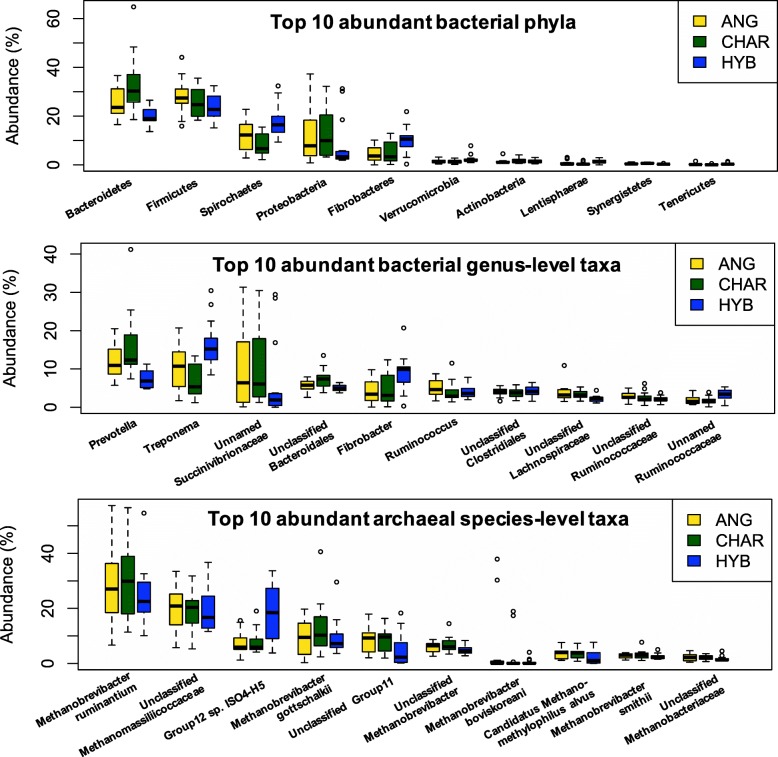
Relative abundances of the most abundant (top ten) rumen microbial taxa (at phylum and genus levels for bacteria and at the species level for archaea) among three beef breeds. ANG, Angus; CHAR, Charolais; HYB, Kinsella composite hybrid

In total, 15 bacterial phyla, 108 bacterial genus-level taxa, and 24 archaeal species-level taxa were identified from T-metatranscriptomes (Additional file 1: Table S1). Among them, 13 bacterial phyla, 66 bacterial genus-level taxa, and 16 archaeal species-level taxa were detected across all samples, confirming reports of a core rumen microbiota [25]. The dominant bacteria phylum was *Bacteroidetes* (26.32 ± 1.34%), followed by *Firmicutes* (25.74 ± 0.91%), *Spirochaetes* (12.81 ± 0.99%), and *Proteobacteria* (11.04 ± 1.54%). At the genus level, *Prevotella* (11.94 ± 0.49%), *Treponema* (11.25 ± 0.95%), unnamed *Succinivibrionaceae* (8.98 ± 1.50%), unclassified *Bacteroidales* (6.05 ± 0.29%), and *Fibrobacter* (6.01 ± 0.64%) were the most abundant bacterial taxa. The rumen archaeal community was dominated by *Methanobrevibacter ruminantium* (27.58 ± 1.82%), unclassified *Methanomassiliicoccaceae* (19.53 ± 1.12%), group 12 sp. ISO4-H5 (*Methanomassiliicoccaceae*-affiliated; 11.05 ± 1.20%), and *Methanobrevibacter gottschalkii* (10.22 ± 1.09%) (Fig. 4 and Additional file 1: Table S1). As rumen digesta samples used in the current study were collected from commercial beef steers under the barley-based high-grain feed, it is unsurprising that the compositional profiles of their rumen digesta are generally comparable to previous described rumen microbial profiles of the cattle fed high grain diet [25].

### Breed effect on the rumen microbiome

**Fig. 5:**
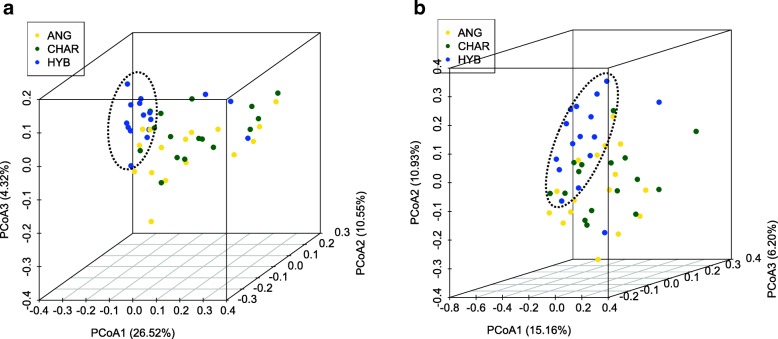
Rumen microbial compositional profiles of three beef breeds visualized using the principal coordinate analysis (PCoA). The PCoA was conducted at the bacterial genus level and at the archaeal species level separately, based on Bray-Curtis dissimilarity matrices. The top three PCoAs were plotted for bacteria (**a**) and archaea (**b**)

The distribution of detected active microbial taxa was different among the three breeds examined (Fig. 4). Although breed did not influence any alpha-diversity indices (*P* > 0.05 by Kruskal-Wallis rank-sum test; Additional file 2: Table S2), the principal coordinate analysis (PCoA) showed that rumen bacterial and archaeal communities in HYB were distinct from those in ANG and CHAR (Fig. 5). Comparisons based on the arcsine square root-transformed relative abundances revealed that around ~ 50% of observed microbial taxa were affected by breed, including 8 bacterial phyla (e.g., *Bacteroidetes* and *Spirochaetes*), 55 taxa at the genus level (e.g., *Prevotella* and *Treponema*), and 10 species-level archaeal taxa (e.g., *Methanomassiliicoccaceae*-affiliated group 12 sp. ISO4-H5 and unclassified *Methanobrevibacter*; Additional file 1: Table S1).

Several biological factors potentially contribute to the rumen microbiota variations observed among breeds. Firstly, we observed significantly different eating frequencies among three breeds: HYB showed lower eat frequency (29.73 ± 1.99 time/day) than that of ANG and CHAR (37.63 ± 1.61 and 36.59 ± 1.56 time/day, respectively; *P* = 6.30e−03) (Table 1). As salivation is enhanced during eating compared to resting [56], lower eating frequencies may lead to lower amounts of saliva produced in HYB, which consequently results in the shift of rumen pH and thus influences the rumen microbiota. Meanwhile, ANG and CHAR had higher feed intake (dry matter intake [DMI]; 10.73 ± 0.27 and 10.33 ± 0.28 kg/day, respectively) than HYB (9.27 ± 0.28 kg/day; *P* = 3.23e−03) (Table 1). It is known that the growth of rumen microbiota is positively correlated with feed intake due to more available substrates and nutrients for the microbial growth [57, 58], and we observed significantly different rumen sizes according to breed (*P* = 1.36e−02) (Table 1). Both feed intake and rumen size have impact on the rumen passage rate [59]. The rumen passage rate could then affect the rumen microbial growth [60, 61], because it is associated with the microbial energy flux (maintenance vs. growth) and microbial generation times [62, 63]. In addition, it has been suggested that the increased rumen passage rate and washout decreased the abundance of rumen methanogens [64], which was further confirmed in a recent study that revealed low CH_4_ yield sheep had smaller rumen size and shorter rumen retention time [65]. Although effects of those biological factors on the rumen microbial growth/abundance have been widely reported as discussed above, how those factors contribute to the variation in microbial composition have not been well described. Therefore, further studies to link those biological factors to microbial compositional profiles are needed, which could help us better understand the breed effect on the rumen microbiota as observed in this study.

**Fig. 6:**
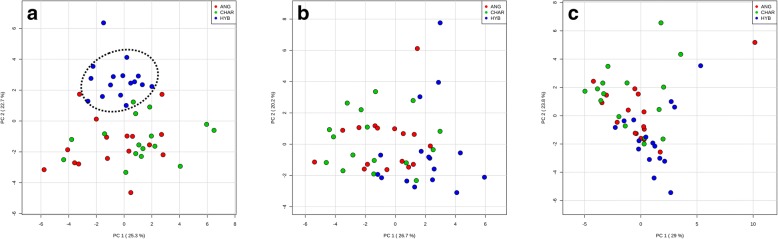
Microbial functional profiles of three beef breeds. PCA for eggNOG functional categories from metagenome (**a**), T-metatranscriptome (**b**), and M-metatranscriptome (**c**) datasets, which were performed based on auto-scaled abundances (cpm) of functional features

Rumen microbiomes from three breeds showed distinguishable functional profiles at the DNA level, especially for the microbiomes from HYB which were distinct from ANG and CHAR (Fig. 6a). Recent studies have reported that gut microbial profiles (estimated based on DNA) of other mammal hosts clustered according to host species [66, 67], suggesting that host genetics could influence functional genetic potentials of the rumen microbiome (although diet is likely to be a major factor also). However, at the transcriptomic level (in both T- and M-metatranscriptomes), differences among three breeds were not obvious (Fig. 6b, c). It has been revealed that dietary intervention significantly alters microbial gene expression profiles without obviously changing the DNA-based microbial profiles in the gut [68], suggesting that metatranscriptomic analysis could better quantify temporal changes of the gut microbiome under environmental change (such as changes in diet). Therefore, the lack of separation of metatranscriptomes among breeds observed in this study may be because the steers were fed the same diet and maintained under the same environmental conditions.

### Differential microbial taxonomic features between RFI groups

As breed-associated differences were observed for ~ 50% of bacteria and archaeal taxa, analyses of the relationships between rumen microbial features and feed efficiency were performed for each breed. Relative abundances of *Firmicutes* (L-RFI: 28.56 ± 1.82% vs. H-RFI: 22.45 ± 2.14%; *P* = 0.042) and *Chloroflexi* (L-RFI: 0.05 ± 0.01% vs. H-RFI: 0.03 ± 0.01%; *P* = 0.046) were different between H- and L-RFI CHAR steers, while no bacterial phylum had statistically different abundances between RFI groups in HYB and ANG. *Chloroflexi* is a phylum of bacteria generally populated by environmentally sampled taxa [69] and has previously been suspected as a transient rumen bacteria [70]. However, recently, two mammalian host-associated genus-level clades were identified within this phylum [69], and taxa belonging to *Chloroflexi* have been detected in the rumen recently [25, 71]. Therefore, their presence or absence in a rumen sample should not be dismissed and they may be considered as ordinarily resident members of the rumen, even though their ecological niche and function in the rumen have not yet been elucidated. Linkages previously identified between rumen *Chloroflexi* and host phenotypes (e.g., milk yield and diet adaptation) [72, 73], in addition to the associations between rumen *Chloroflexi* and feed efficiency identified in the current study, highlight the importance of defining its role in the rumen in future studies. At the bacterial genus level, 22 (e.g., unnamed *Bacteroidales* and *Butyrivibrio*), one (unnamed RF16), and 16 genus-level taxa (e.g., unclassified *Clostridiales* and unnamed *Ruminococcaceae*) were significantly differentially abundant between H- and L-RFI steers in HYB, ANG, and CHAR, respectively (*P* < 0.05; Table 3). For archaea, differences in abundance of *Methanobrevibacter smithii* and four taxa (unclassified *Methanomassiliicoccaceae*, unclassified *Methanobrevibacter*, unclassified group 11, and *Methanomethylophilus alvus*) were detected between H- and L-RFI steers (*P* < 0.05) in HYB and CHAR, respectively, but no differential archaeal taxa were detected between RFI groups in ANG (Table 3). Meanwhile, H- and L-RFI HYB steers statistically differed in bacterial community diversity (*P* = 0.04) (as calculated by the Shannon index). For CHAR steers, two RFI groups had significantly different inverse Simpson (*P* = 0.03) and Simpson evenness (*P* = 0.03) of archaeal communities, as well as Shannon evenness of bacteria communities (*P* = 0.03) (Table 4).

**Table 3:** Relative abundances of differential microbial taxa between RFI groups in three beef breeds

	Taxon	H-RFI	L-RFI	*P* value^1^
		(mean ± SEM) (%)	(mean ± SEM) (%)	
Angus				
Genus level	Unnamed RF19	0.49 ± 0.08	1.31 ± 0.32	0.045
Charolais				
Phylum level	*Firmicutes*	22.45 ± 2.14	28.56 ± 1.82	0.042
Phylum level	*Chloroflexi*	0.03 ± 0.01	0.05 ± 0.01	0.046
Genus level	Unclassified *Clostridiales*	3.05 ± 0.36	4.38 ± 0.25	0.008
Genus level	Unnamed *Ruminococcaceae*	1.20 ± 0.34	2.15 ± 0.32	0.040
Genus level	Unnamed S24-7	0.76 ± 0.20	1.84 ± 0.44	0.025
Genus level	*Succiniclasticum*	0.34 ± 0.15	0.91 ± 0.10	0.004
Genus level	Unnamed *Mogibacteriaceae*	0.38 ± 0.06	0.58 ± 0.05	0.023
Genus level	*Moryella*	0.29 ± 0.05	0.56 ± 0.04	0.002
Genus level	Unclassified *Clostridia*	0.20 ± 0.02	0.32 ± 0.04	0.012
Genus level	CF231	0.09 ± 0.02	0.21 ± 0.03	0.009
Genus level	Unclassified *Lachnospiraceae*	0.07 ± 0.01	0.11 ± 0.01	0.042
Genus level	p-75-a5	0.05 ± 0.01	0.10 ± 0.02	0.023
Genus level	Unclassified *Mogibacteriaceae*	0.05 ± 0.01	0.08 ± 0.01	0.049
Genus level	R4-45B	0.01 ± 0.01	0.04 ± 0.01	0.019
Genus level	*Blautia*	0.004 ± 0.001	0.013 ± 0.003	0.004
Genus level	*Adlercreutzia*	0.004 ± 0.001	0.007 ± 0.001	0.046
Genus level	Unclassified *Christensenellaceae*	0.004 ± 0.001	0.007 ± 0.001	0.043
Genus level	Unclassified *Anaerolineae*	0.003 ± 0.000	0.005 ± 0.001	0.018
Species level	Unclassified *Methanomassiliicoccaceae*	23.41 ± 1.31	14.65 ± 2.96	0.014
Species level	Unclassified *Methanobrevibacter*	5.48 ± 0.65	8.24 ± 1.05	0.033
Species level	Unclassified group11	10.72 ± 0.86	6.22 ± 1.69	0.019
Species level	*Candidatus Methanomethylophilus alvus*	4.46 ± 0.37	2.68 ± 0.75	0.027
Kinsella composite hybrid				
Genus level	Unclassified *Bacteroidales*	1.04 ± 0.13	1.62 ± 0.09	0.003
Genus level	Unclassified *Bacteroidetes*	0.86 ± 0.16	1.73 ± 0.15	0.001
Genus level	*Butyrivibrio*	0.97 ± 0.12	1.58 ± 0.18	0.009
Genus level	Unnamed *Victivallaceae*	0.77 ± 0.17	1.70 ± 0.21	0.006
Genus level	*Desulfovibrio*	0.21 ± 0.03	0.43 ± 0.05	0.002
Genus level	Unnamed *Mogibacteriaceae*	0.24 ± 0.02	0.36 ± 0.05	0.042
Genus level	Unclassified *Clostridia*	0.20 ± 0.02	0.29 ± 0.02	0.009
Genus level	Unnamed *Christensenellaceae*	0.07 ± 0.01	0.25 ± 0.10	0.036
Genus level	Unclassified *Paraprevotellaceae*	0.18 ± 0.03	0.12 ± 0.01	0.015
Genus level	*Shuttleworthia*	0.26 ± 0.09	0.03 ± 0.01	0.008
Genus level	Unnamed *Paraprevotellaceae*	0.19 ± 0.04	0.10 ± 0.01	0.045
Genus level	Unclassified *Aeromonadales*	0.15 ± 0.07	0.01 ± 0.00	0.027
Genus level	Unnamed *Spirochaetaceae*	0.04 ± 0.01	0.10 ± 0.02	0.015
Genus level	*Desulfobulbus*	0.10 ± 0.03	0.03 ± 0.01	0.009
Genus level	Unclassified *Verrucomicrobia*	0.04 ± 0.01	0.07 ± 0.01	0.045
Genus level	Unnamed R4-45B	0.02 ± 0.01	0.07 ± 0.02	0.008
Genus level	*Mitsuokella*	0.05 ± 0.01	0.01 ± 0.00	0.029
Genus level	L7A_E11	0.01 ± 0.00	0.04 ± 0.01	0.006
Genus level	*Roseburia*	0.04 ± 0.01	0.01 ± 0.00	0.020
Genus level	*Blautia*	0.01 ± 0.00	0.04 ± 0.01	0.035
Genus level	Unclassified *Lentisphaeria*	0.01 ± 0.00	0.03 ± 0.01	0.005
Genus level	Unclassified *Desulfovibrionaceae*	0.01 ± 0.00	0.02 ± 0.01	0.039
Species level	*Methanobrevibacter smithii*	2.04 ± 0.17	2.95 ± 0.38	0.040

^1^*P* values were calculated between H- and L-RFI groups using *t* test within each breed, based on arcsine square root-transformed relative abundances

**Table 4:** Comparisons of alpha-diversity indices^1^ between beef cattle with different RFI values

	Angus (*n* = 16)	Charolais (*n* = 16) (mean ± SEM)	Kinsella composite hybrid (*n* = 16)
Bacteria			
Chao1			
L-RFI	317.03 ± 11.83	319.87 ± 12.95	381.88 ± 37.60
H-RFI	337.64 ± 21.82	326.69 ± 16.70	358.74 ± 27.93
*P* value^2^	NS	NS	NS
Shannon evenness			
L-RFI	0.57 ± 0.01	0.59 ± 0.01	0.57 ± 0.01
H-RFI	0.58 ± 0.01	0.55 ± 0.02	0.54 ± 0.01
*P* value^2^	NS	0.03	NS
Simpson evenness			
L-RFI	0.05 ± 0.00	0.05 ± 0.01	0.05 ± 0.00
H-RFI	0.05 ± 0.00	0.04 ± 0.00	0.04 ± 0.00
*P* value^2^	NS	NS	NS
Shannon index			
L-RFI	3.17 ± 0.05	3.28 ± 0.06	3.21 ± 0.04
H-RFI	3.18 ± 0.06	3.06 ± 0.10	3.01 ± 0.07
*P* value^2^	NS	NS	0.04
Inverse Simpson			
L-RFI	12.26 ± 0.96	13.89 ± 1.22	13.18 ± 0.74
H-RFI	12.78 ± 0.97	10.96 ± 1.31	10.53 ± 1.14
*P* value^2^	NS	NS	NS
Archaea			
Chao1			
L-RFI	25.88 ± 1.03	28.06 ± 2.36	24.17 ± 0.95
H-RFI	24.25 ± 1.22	24.65 ± 1.38	25.31 ± 1.32
*P* value^2^	NS	NS	NS
Shannon evenness			
L-RFI	0.64 ± 0.02	0.62 ± 0.02	0.66 ± 0.02
H-RFI	0.64 ± 0.02	0.66 ± 0.01	0.65 ± 0.01
*P* value^2^	NS	NS	NS
Simpson evenness			
L-RFI	0.22 ± 0.02	0.20 ± 0.01	0.24 ± 0.02
H-RFI	0.23 ± 0.02	0.26 ± 0.02	0.24 ± 0.01
*P* value^2^	NS	0.03	NS
Shannon index			
L-RFI	2.00 ± 0.07	1.95 ± 0.06	2.07 ± 0.07
H-RFI	1.98 ± 0.07	2.07 ± 0.04	2.05 ± 0.03
*P* value^2^	NS	NS	NA
Inverse Simpson			
L-RFI	5.21 ± 0.51	4.74 ± 0.37	5.67 ± 0.46
H-RFI	5.19 ± 0.50	5.93 ± 0.36	5.48 ± 0.14
*P* value^2^	NS	0.03	NS

*NS* not significant with *P* value not less than 0.05

^1^To make alpha-diversity indices comparable among samples, the number of sequences per sample was normalized to the lowest reads number for bacteria (*n* = 274,885) and archaea (*n* = 4263), respectively

^2^*P* values were obtained between H- and L-RFI groups within each breed using the Kruskal-Wallis rank-sum test

Differential taxonomic features between H- and L-RFI groups were not consistent among three breeds, except for four differential bacterial genus-level taxa in HYB and CHAR (*Blautia*, unclassified *Clostridia*, unnamed *Mogibacteriaceae*, and unnamed R4-45B). Although these bacterial taxa were low abundant in the rumen (< 0.5%), it is notable that they all showed higher abundances in L-RFI animals than in H-RFI individuals in both HYB and CHAR (Table 3). *Blautia* members are ubiquitously distributed in mammal gut with low abundance [74]. They have been reported to provide energy to hosts from the fermentation of polysaccharides that other microbial taxa cannot [75], and thus a higher abundance of *Blautia* may extend the rumen metabolic capacity for steers with high feed efficiency. In addition, members of *Blautia* (such as *Blautia hydrogenotrophica*), have the capacity to consume H_2_ and produce acetate through acetogenesis [76]. Therefore, the increased abundance of *Blautia* indicates possible higher acetogenesis in L-RFI animals, leading to greater competition with rumen methanogens. More acetates rather than CH_4_ could be generated during removal of H_2_ from the rumen in L-RFI individuals, leading to lower energy waste. To find experimental evidence for our inference mentioned above, providing beef cattle with *Blautia* cultures and then testing whether it could improve the feed efficiency of beef cattle and alter rumen microbial functions should be considered as a future study direction. A *Mogibacteriaceae*-affiliated unnamed genus has already been reported to be associated with feed efficiency in beef cattle with multiple genetic backgrounds [9], but scarce information is available to define its functions in the rumen. Abundances of members in this family were negatively correlated with body mass index (BMI) in humans [77, 78], suggesting the higher abundance of *Mogibacteriaceae* in L-RFI individuals may correspond to a leaner body type and further correspond to a higher protein deposition in individuals with high feed efficiency.

While 48 steers involved in this study received identical diet and were raised under the same environmental conditions, different rumen microbial communities were distinguishable among the different breeds studied and unique differential taxonomic profiles were observed between RFI groups within each breed. This suggests that several rumen microorganisms belonging to different taxonomic groups may share similar ecological niches in different hosts, utilizing similar substrates and producing similar products in the rumen. Indeed, previous studies in humans have demonstrated that functional profiles of the microbiome are more conserved than the taxonomic composition at certain body sites [19, 79]. In ruminants, it has been observed that even when rumen microbiomes are dissimilar at the taxonomic level, they can share similar metabolic functions [80]. Furthermore, two recent studies have shown that methane emissions and RFI are more associated with rumen microbial functional profiles than taxonomic profiles [24, 81]. Collectively, this suggests that only analyzing the rumen microbial communities may be not sufficient to discover real biological linkages between the rumen microbiome and feed efficiency. Therefore, it is necessary to further investigate how rumen microbial functional features contribute to the variation in feed efficiency.

### Differential microbial functions between H- and L-RFI steers

In ANG, RFI had no effect on functional categories identified from metagenomes and T-metatranscriptomes, while “RNA processing and modification” showed higher abundance in M-metatranscriptomes of L-RFI animals than that of H-RFI ones (*P* = 0.021). For CHAR, two functional categories “Cell cycle control, cell division, chromosome partitioning” and “Secondary metabolites biosynthesis, transport and catabolism” were more abundant in H-RFI animals than in L-RFI animals at the genomic level (*P* = 0.008 and 0.033, respectively). In T- and M-metatranscriptomes, four and two functional categories were differentially abundant between RFI groups, respectively. Interestingly, “Translation, ribosomal structure and biogenesis” and “Transcription” had higher expression levels in H-RFI animals from both T- and M-metatranscriptomes (*P* < 0.05; Table 5). For HYB steers, “Intracellular trafficking, secretion, and vesicular transport” was higher abundant in H-RFI steers than in L-RFI steers at the DNA level (*P* = 0.001). “Cell motility” was more abundant at the transcriptomic level in both T- and M-metatranscriptomes (*P* = 0.044 and 0.013, respectively). “Nucleotide transport and metabolism” and “Cytoskeleton” only showed differential abundances in T-metatranscriptomes (*P* = 0.010 and 0.036, respectively).

**Table 5:** Abundances of differential functional categories between RFI groups in three beef breeds

	Functional category	H-RFI	L-RFI	*P* value
		(mean ± SEM; cpm)	(mean ± SEM; cpm)	
Angus				
M-metatranscriptome	RNA processing and modification	49.84 ± 10.56	195.48 ± 103.74	0.021
Charolais				
Metagenome	Cell cycle control, cell division, and chromosome partitioning	12,909.00 ± 347.45	11,589.90 ± 301.67	0.008
Metagenome	Secondary metabolites biosynthesis, transport, and catabolism	12,971.28 ± 700.02	11,296.28 ± 508.41	0.033
T-metatranscriptome	Translation, ribosomal structure, and biogenesis	96,639.54 ± 3962.26	84,353.42 ± 3284.80	0.026
T-metatranscriptome	Transcription	50,431.27 ± 982.96	48,084.54 ± 739.42	0.025
T-metatranscriptome	Coenzyme transport and metabolism	32,933.44 ± 1089.03	35,990.82 ± 1219.48	0.046
T-metatranscriptome	Chromatin structure and dynamics	874.42 ± 160.48	1409.80 ± 122.53	0.041
M-metatranscriptome	Translation, ribosomal structure, and biogenesis	92,501.13 ± 4666.25	72,050.06 ± 3878.38	0.001
M-metatranscriptome	Coenzyme transport and metabolism	33,326.68 ± 825.36	38,128.97 ± 1781.97	0.014
Kinsella composite hybrid				
Metagenome	Intracellular trafficking, secretion, and vesicular transport	16,275.15 ± 367.52	14,238.87 ± 417.81	0.001
T-metatranscriptome	Cell motility	24,372.19 ± 1793.06	33,107.68 ± 5178.07	0.044
T-metatranscriptome	Nucleotide transport and metabolism	28,338.08 ± 1255.51	24,255.68 ± 1009.29	0.010
T-metatranscriptome	Cytoskeleton	4102.77 ± 1936.53	4132.47 ± 3014.61	0.036
M-metatranscriptome	Cell motility	24,493.50 ± 1808.59	37,334.38 ± 6269.31	0.013

*cpm* counts per million reads

*P* values were obtaining between H- and L-RFI steers using DESeq2 within each breed

**Fig. 7:**
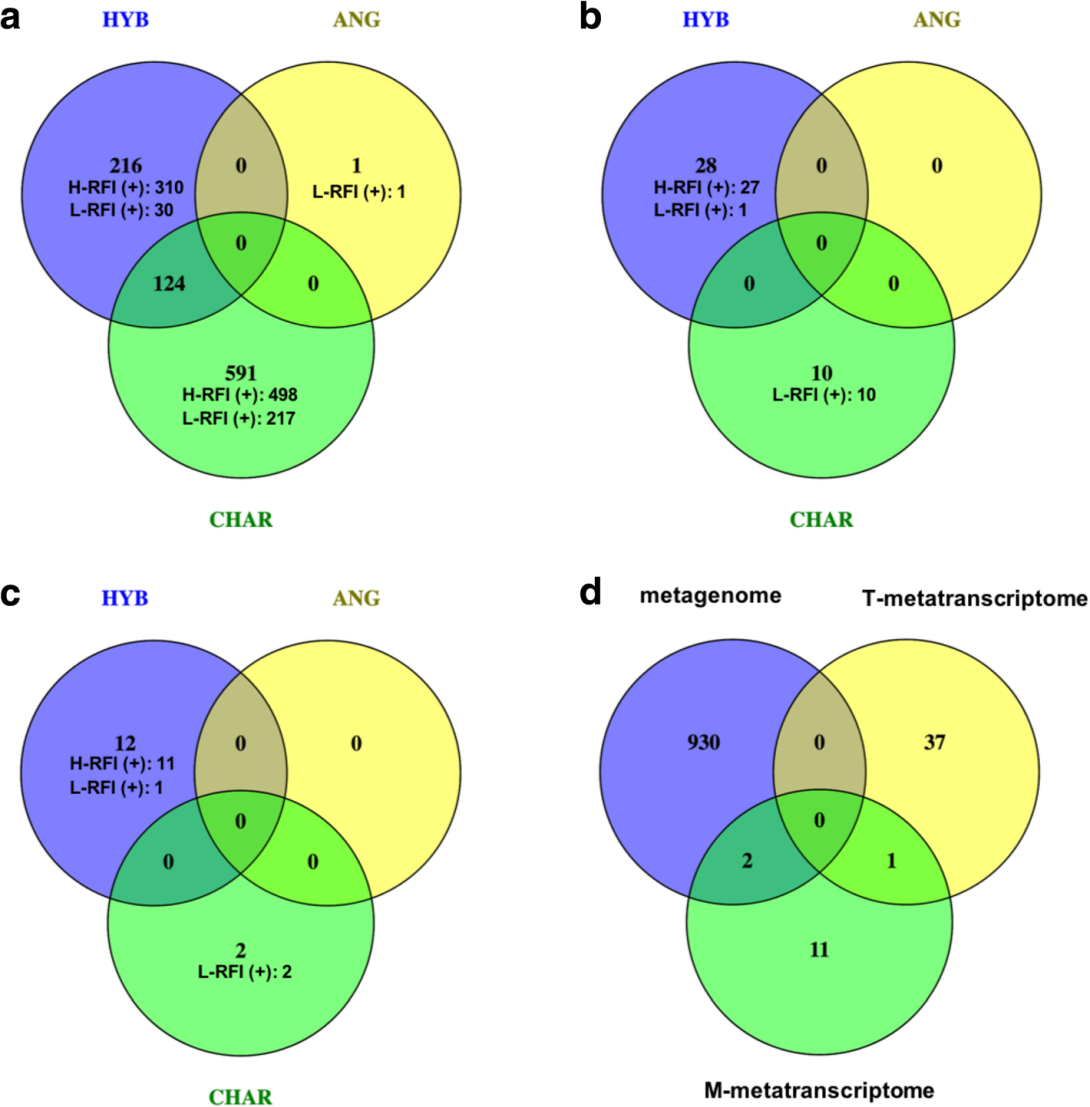
Identified differential genes/transcripts between H- and L-RFI groups from metagenome (**a**), T-metatranscriptome (**b**), and M-metatranscriptome (**c**) datasets, as well as differential genes/transcripts between RFI groups in all three datasets (**d**). H-RFI (+) and L-RFI (+) represents the number of genes/transcripts enriched in H-RFI and L-RFI animals, respectively

Comparative analysis of metagenomes revealed 932 genes (range of gene coverage 40–6067×) with differential abundances between H- and L-RFI animals from metagenomes: 591 genes in CHAR, 216 genes in HYB, and one gene in ANG, with 124 genes overlapped in both CHAR and HYB. When compared T-metatranscriptomes, there were 38 differentially expressed genes (range of gene coverage 4–186×) between RFI groups (28 in HYB, ten in CHAR, and none in ANG). From the comparison of M-metatranscriptomes, RFI had effects on 14 expressed genes (12 in HYB and two in CHAR; range of gene coverage 57–976×) (Fig. 7a–c). It is notable that only three differential genes were detected between H- and L-RFI steers at both DNA and RNA levels: two were found in both metagenomes and M-metatranscriptomes (genes coding 2,3-bisphosphoglycerate-independent phosphoglycerate mutase and coding fumarate reductase/succinate dehydrogenase flavoprotein domain protein) and one was found in both T- and M-metatranscriptomes (gene coding phosphoketolase) (Fig. 7d).

Recent studies suggest that rumen microbiomes of H-RFI animals have more diverse functional potentials [11] and higher activities [24] than those of L-RFI individuals. Those findings are further confirmed in the present study with the higher abundances of differential genes/transcripts observed in H-RFI steers than in L-RFI ones (Fig. 7a–c). This suggests that rumen microorganisms of inefficient individuals are capable of fermenting a wider range of substrates and can generate more products. However, these products may be harmful, of little use or exceed the absorbing capacity of the host (as determined by host genetics), and lead to low feed efficiency. Conversely, efficient cattle have relatively simpler rumen microbial functions and lower activities, possibly generating more specific products that can be more efficiently absorbed and utilized by the host.

In the present study, although some microbial genes were differentially abundant between RFI groups in both CHAR and HYB metagenomes (Fig. 7a), no functional category (at the DNA or RNA level) or expressed gene was found to be different between H- and L-RFI steers across all three breeds (Table 5 and Fig. 7b, c). This suggests that different rumen microbiome-host interaction patterns determine the feed efficiency performance in each beef cattle breed. For example, from all three sequencing datasets, we observed only few differential microbial features (at both compositional and functional levels) between H- and L-RFI steers in ANG, suggesting that the rumen microbiome in ANG may only have a small contribution to the RFI variations observed. In contrast, many more compositional and functional features of the rumen microbiome in HYB and CHAR were associated with host RFI performance. Considering the different genetic backgrounds (different genotypes) among three breeds, further studies to explore the interactions between the rumen microbiome and host genotypes are needed to better understand how these interactions may affect feed efficiency in beef cattle.

## Conclusions

The current study has not only addressed several critical methodological questions in terms of rumen meta-omics, but also demonstrates the associations of the rumen microbiome with host breed and feed efficiency. Our results suggest that metatranscriptomics may be a more powerful approach for associating rumen microorganisms with host performances. In addition, although the mRNA enrichment increased the sequencing depth of mRNA and enhanced the resolution of metatranscriptomics on the functional analysis, comparison of total-RNA-based and mRNA-enriched metatranscriptomes revealed potential biases in the estimation of some gene expression levels within mRNA-enriched metatranscriptomes. Therefore, we suggest that mRNA-enriched metatranscriptomics are best used for the study of specific genes and/or metabolic pathways especially with low expression levels, while total RNA-based metatranscriptomics are best applied for linking overall compositional and functional profiles of the rumen microbiome to host phenotypes. It should be noted that extremely low abundant and lowly expressed genes may not be detected due to insufficient sequencing depth of current metagenome and metatranscriptome datasets. However, to date, it is not yet clear what sequencing depth is necessary to comprehensively capture the entire rumen microbial genes and/or transcripts. Benchmark studies with gold standard reference datasets are required to establish a standard protocol with reliable criteria for rumen metagenomics and metatranscriptomics. Furthermore, a large proportion of metagenome contigs could not be annotated based on existing databases (40–50%), highlighting the need to characterize more microbial genomes from rumen and expand the coverage of the rumen microbiome in existing databases. Supporting this, the Hungate1000 collection combined with earlier sequencing efforts has resulted in the sequencing of over 500 cultured bacteria and archaea from the rumen [82] and ongoing efforts to reconstruct additional genomes from metagenomic data are likely to contribute to this resource [49]. These rumen-specific reference genomes will enhance the power of rumen metagenomic and metatranscriptomic analysis and better guide the date interpretation in future studies.

Taxonomic analysis of total RNA-based metatranscriptomics revealed distinguishable active rumen microbiota, and metagenomic data revealed different functional genetic potentials according to host genetic background. These breed-associated differences represent potential superiorities of each breed, which could further be applied to manipulate the rumen microbiome through selective breeding of the hosts. In contrast, the actual activities of the rumen microbiome were less impacted by host genetics but were more sensitive to environmental factors. Several differential microbial features between RFI groups were detected within each breed, including active bacterial and archaeal taxa, alpha-diversity indices of microbial communities, functional categories, and genes (at both DNA and RNA levels). These results extend our understanding on associations between the rumen microbiome and feed efficiency at multiple genetic levels in diverse beef cattle breeds. Most of differential microbial features between H- and L-RFI steers were distinct among three breeds, suggesting there are host and microbiome interactions in the rumen contributing to the variation in feed efficiency.

## Additional files

Additional file 1: **Table S1.** Rumen microbial compositional profiles among three beef breeds. (XLSX 50 kb)

Additional file 2: **Table S2.** Comparisons of alpha-diversity indices among three beef breeds. (XLSX 38 kb)
